# T cell, M2 macrophage infiltration and collagen, fibronectin, VCAM-1 expression portend poor outcome in lupus nephritis

**DOI:** 10.3389/fimmu.2026.1802673

**Published:** 2026-08-12

**Authors:** Sen Hee Tay, Anto Sam Crosslee Louis Sam Titus, Vinaika Maruvada, Bernett Teck Kwong Lee, Gek Cher Chan, Alvin Seng Cheong Wong, Jiacai Cho, Thomas Paulraj Thamboo, Chandra Mohan

**Affiliations:** 1 Division of Rheumatology and Allergy, National University Hospital, Singapore, Singapore; 2 Department of Medicine, Yong Loo Lin School of Medicine, National University of Singapore, Singapore, Singapore; 3 Department Biomedical Engineering, University of Houston, Houston, TX, United States; 4 Centre for Biomedical Informatics, Lee Kong Chian School of Medicine, Nanyang Technological University, Singapore, Singapore; 5 Singapore Immunology Network, Agency for Science, Technology and Research, Singapore, Singapore; 6 Division of Nephrology, National University Hospital, Singapore, Singapore; 7 Department of Haematology-Oncology, National University Cancer Institute, Singapore, Singapore; 8 Department of Pathology, National University Hospital, Singapore, Singapore

**Keywords:** Bayesian network analysis, lupus nephritis, macrophages, proteomics, treatment outcome

## Abstract

**Introduction:**

Despite advances in lupus nephritis (LN) management, its heterogenous nature poses challenges in predicting treatment response. Multiplexed spatial proteomics offers a potential solution to dissect the heterogeneity of treatment response in LN.

**Methods:**

Renal response was assessed in 16 LN patients undergoing physician-directed induction therapy. Baseline biopsies from these patients were subjected to 34-plex spatial proteomics. A total of 53 glomerular and 49 tubulointerstitial regions of interest from these LN patients and renal cell carcinoma (RCC) controls were analyzed for spatial predictors of treatment response.

**Results:**

There were increased glomerular and tubulointerstitial immune infiltrates in LN kidneys compared to RCC controls, including CD45RO, HLA-DR, and CD68-positive cells. Interestingly, there was increased glomerular and interstitial collagen I, collagen IV deposition, and fibronectin extracellular matrix (ECM) proteins in class V compared to class IV LN. Glomerular and tubulointerstitial immune infiltrates and fibrosis in pre-treatment biopsies predicted non-response (NR) to immunosuppressive therapy, including leukocytes expressing CD45RO, HLA-DR, CD4, CD14, and CD163 and collagen I, collagen IV, and fibronectin expression. Increased tubular and parietal epithelial cell expression of the activation/injury marker VCAM-1 also marked treatment NR.

**Discussion:**

As defined by multiplexed spatial proteomics, increased infiltration of activated T cells and CD163^+^ M2 macrophages, glomerular and tubulointerstitial fibrosis, and heightened expression of the injury marker VCAM-1 portend poor response to immunosuppressive treatment in LN.

## 1 Introduction

Lupus nephritis (LN) is a heterogeneous disease, and its response to treatment can vary substantially among patients. The choice of therapy depends on several clinical factors such as age, ancestry, history of LN in the past, renal function, histological class, activity, chronicity, medical comorbidities, expected tolerance, and adherence to treatment (1, 2). The recent addition of belimumab, obinutuzumab, and voclosporin to the therapeutic armamentarium of LN has raised questions of how best to apply them (3, 4). One of the most pressing issues has been identifying a LN patient who needs more than standard of care and whether belimumab, obinutuzumab, or voclosporin would be the best for the individual (3, 4). In addition, whether to adopt a step-down (“hit hard and early”) or step-up (“wait and see”) approach in the treatment algorithm has not been studied in LN (1). Despite the broad recognition of the International Society of Nephrology/Renal Pathology Society (ISN/RPS) classification system and the unequivocal value of histopathological findings to confirm the diagnosis, inform treatment decisions, and prognosis, insights from molecular studies such as serum and urinary proteome have not been incorporated in routine clinical practice for personalized therapeutic strategies (2, 5, 6). Molecular profiling of LN patients can hopefully assist in stratification to allow personalization of treatment (1). In addition, machine learning approaches are likely to transform the diagnosis and management of LN in the coming decades (6).

Proteomics discovery methods are commonly used to identify biomarkers in LN (7). Diverse biological fluids have been used for proteomic studies, including plasma, serum, and urine (7, 8). Urinary proteomics seems to be particularly promising as up to 70% of peptides and proteins are of renal origin, and the proximity to the disease pathology has allowed the identification of adhesion molecules, autoantibodies, chemokines, complement proteins, and cytokines as potential biomarkers of LN (9). However, urine as a biological sample for biomarker identification in LN can be confounded by renal filtration and leakage of proteins from blood (10). In addition, analysis of urinary proteomics does not provide the spatial context that is necessary to determine cellular proximity and interactions between cell types (11). In this study, we performed imaging mass cytometry (IMC)-based spatial proteomics and machine learning on kidney biopsy samples to dissect the heterogeneity of the treatment response in LN and to identify the pre-treatment molecular predictors of response to immunosuppressive therapy.

## 2 Materials and methods

### 2.1 Subjects

We recruited SLE patients with biopsy-proven pure class IV and pure class V LN from National University Hospital (NUH), Singapore. All SLE patients had their first kidney biopsy performed for diagnosis retrieved from the pathology archive for analysis. Patients with previous nephrectomies for renal cell carcinoma (RCC) were recruited as disease controls. Normal adjacent tissue in nephrectomy samples from RCC patients was used for comparison. Kidney biopsies were read by an experienced renal pathologist (T.P.T.) and classified according to the ISN/RPS criteria and modified National Institutes of Health (NIH) activity and chronicity indices (5). The proposed modified National Institute of Health indices were used to report the overall activity and chronicity (5). Choice of induction therapy was mainly based on the best clinical practices of the time, including cyclophosphamide, mycophenolate mofetil/mycophenolate sodium, azathioprine, cyclosporine, rituximab, and tacrolimus (with the first two being the most common). Renal response was assessed at 12 months after induction therapy with definitions similar to the those provided by *Kidney Disease: Improving Global Outcomes* guidelines for LN (12). Complete response (CR) was defined as estimated glomerular filtration rate (eGFR) within the normal range or not less than 85% of baseline and 24-h urinary protein/urinary creatinine ratio (UPCR) ≤0.5 mg/mg. Partial response (PR) was defined as normal eGFR or not less than 85% of baseline and ≥50% reduction in 24-h UPCR to subnephrotic range levels. “Baseline” kidney function refers to the level before disease flare (12). No response (NR) was defined as failure to achieve PR or CR. Written informed consent was obtained from all participants. The study was approved by National Healthcare Group Domain Specific Review Board E (reference number: 2020/00644).

### 2.2 Tissue processing and selection of regions of interest

From each formalin-fixed paraffin-embedded biopsy block, two consecutive 5-μm-thick sections were mounted on separate glass slides. One was stained with hematoxylin and eosin for histopathological assessment and selection of regions of interest (ROIs), while the adjacent section underwent 35-plex IMC staining using a combination of DNA dye and 34 metal-tagged antibodies for interrogating resident renal cells, immune cells, extracellular matrix (ECM) proteins, epithelial-to-mesenchymal plasticity (EMP) regulators, and functional status proteins. Glomerular and tubulointerstitial ROIs representative of the biopsy histopathology were selected for IMC spatial proteomics. Renal structural markers and extracellular matrix antibodies were validated by conventional immunohistochemistry, whereas immune cell markers were validated on spleen tissue using IMC (11). In total, 102 ROIs were examined, comprising 53 glomerular ROIs (300 × 300 μm, including the periglomerular rim) and 49 tubulointerstitial ROIs (200 × 400 μm), selected from eight class IV LN, eight class V LN, and three RCC patients.

### 2.3 Imaging mass cytometry staining, acquisition, and signal pre-processing

Imaging mass cytometry was performed as detailed in our earlier report (11). The sectioned slides were stored at 4°C until ready to be processed for IMC; the sections were then deparaffinized and rehydrated. Antigen retrieval was performed in Tris buffer (pH 9), followed by blocking with bovine serum albumin in phosphate-buffered saline (PBS). Antibodies were conjugated to different metal tags using the Maxpar kit (Fluidigm) according to the manufacturer’s instructions, and the sections were stained overnight at 4°C with a cocktail of antibodies. The slides were then washed with PBS/Tween to remove unbound antibodies, and the tissue was then ablated with Nd: YAG 213 nm laser on Hyperion Imaging System coupled to a Helios CyTOF mass cytometer (Fluidigm).

Data acquisition generated MCD files, which were inspected in MCD Viewer. TIFF images were extracted and processed in Python (v3.9) using *imctools* to remove hot pixels, based on outliers detected in the Xe^134^ antibody-free background channel. A 5 px × 5 px median filter was applied for smoothing the hot pixels. Boxplots were used to determine the top 10% of non-zero pixel intensities to set positive signal ranges. Pattern-aware sampling was applied to reduce salt-and-pepper noise, and clusters smaller than five connected pixels were removed as artifacts because cells are anticipated to be much larger clusters of connected pixels. Pixel-wise correlation analysis was performed for each channel pair to detect potential channel spillover. Xe134 served as a negative control, while Ir191 and Ir193 (both DNA markers) served as positive controls. Panel optimization steps were repeated until spillover artifacts were eliminated.

### 2.4 Data analysis

IMC data after pre-processing were used to generate TIFF stacks which were analyzed in ImageJ by splitting into individual channels (markers). Min–max thresholding was performed to reduce background noise and improve the signal–noise ratio. Combinations of markers were generated with *Merge channels* function in ImageJ with pseudo-colors to visualize the spatial expression of the markers in the ROI. *Watershed* algorithm in ImageJ was used to separate joint cells. *Analyze particles* function in ImageJ was used to obtain the cell counts in the cell-specific marker channels. The cell counts were normalized by the area of the ROI to generate cell counts per square millimeter of tissue, and gray-scale pixel values were used to determine the expression levels of markers in the ROI.

Bayesian network analysis was performed using BayesiaLab software to investigate relationships between treatment outcomes, clinical variables, pathology indices, and renal protein biomarkers expressed in the glomerular and tubulointerstitial compartments. Since IMC proteomics generated a high-dimensional dataset with a large number of spatial and clinical variables relative to the available cohort size, this exploratory unsupervised machine learning approach was used to identify probabilistic dependency patterns and conditional relationships among clinical, pathological, treatment, and IMC-derived variables rather than to develop a predictive model. Demographic features, clinical measures, pathology indices, treatment response, and compartment-specific renal protein expression were included in the analysis. Missing values were imputed prior to analysis using class-specific imputation within each lupus nephritis group, with median imputation applied for continuous variables and mode imputation applied for categorical variables. Two separate unsupervised networks were generated using the EQ algorithm: one for the glomerular proteome and one for the tubulointerstitial proteome. In these networks, node size reflects the relative impact of the node, while arcs represent probabilistic conditional dependencies between variables. Arc thickness corresponds to the relative strength of the probabilistic dependency, and arc color reflects the directionality of the Pearson correlation coefficient used for visualization purposes (blue = positive, red = negative).

To address the non-independence of multiple ROIs originating from the same patient and to evaluate the influence of treatment heterogeneity on treatment response, ROI-level multinomial regression analyses were additionally performed. Separate models were fitted for glomerular and tubulointerstitial ROIs. Treatment response status was modeled as the dependent variable, with response categories encoded as 0 = no response (NR), 1 = complete response (CR), and 2 = partial response (PR), with NR serving as the reference category. Gender was encoded as 1 = male and 2 = female, while lupus nephritis class was encoded as 1 = class IV and 2 = class V, with male sex and class IV serving as the reference categories, respectively. These categorical encodings were consistently used across both Bayesian network and multinomial regression analyses. Modified NIH activity index, modified NIH chronicity index, and LN class were included as core adjustment variables in all multinomial regression models due to their established pathological relevance to renal outcome. Because the simultaneous inclusion of all IMC biomarkers and treatment variables would substantially increase model dimensionality relative to the cohort size and lead to model instability and overfitting, each renal protein biomarker was evaluated individually in separate models. Similarly, induction therapy variables were incorporated one at a time rather than simultaneously in a single multivariable model. This simplified modeling strategy was selected to permit the exploratory assessment of marker–response associations while maintaining statistical stability within the limited sample size. Given the exploratory nature of the study, limited cohort size, and high dimensionality of the IMC dataset, *p <*0.05 was considered indicative of trend-level associations. Bayesian network analyses were performed separately at the patient level using aggregated ROI measurements to identify broader conditional dependency patterns among clinical, pathological, and spatial proteomic variables. These complementary patient-level Bayesian network analyses and ROI-level multinomial regression analyses were used to identify both global dependency structures and localized marker–response associations within the spatial proteomic dataset.

### 2.5 Comparison of fibrosis-related gene expression between classes IV and V LN

A dataset from Almaani et al. (GSE127797) that contained the required sample types was used (13). Renal gene expression microarray data in the form of normalized robust multi-array average expression values were obtained from GEO and analyzed in Bioconductor (R version 4.1.2). Protein-coding genes for collagen I, collagen IV, and fibronectin were obtained from HUGO Gene Nomenclature Committee; gene groups and expression data were analyzed using hierarchical cluster analysis and visualized as a heat map.

### 2.6 Statistics

Data were analyzed using non-parametric Mann–Whitney *U*-test and Spearman’s rank correlation coefficient, while graphs were plotted using GraphPad Prism 9. Two-tailed *p*-values less than 0.05 were deemed statistically significant, where * denotes *p <*0.05, ** denotes *p <*0.01, and *** denotes *p <*0.001.

## 3 Results

### 3.1 Demographics and clinical characteristics

A total of eight class IV LN, eight class V LN, and three RCC patients were recruited, with data on demographics and key clinical characteristics presented in **Tables 1** and **2**. The majority of SLE patients were female (68.8%) and of Chinese ethnicity (81.3%). Patients with class IV LN were significantly younger [37.0 (25.3–46.5) vs. 53.0 (47.8–63.3), *p* = 0.027] and had higher activity index [7.0 (5.3–8.8) vs. 0.0 (0.0–0.3), *p* < 0.001] but comparable chronicity index [1.0 (0.0–3.0) vs. 4.0 (1.5–4.0), *p* > 0.05] compared to class V LN. Glomerular and tubulointerstitial regions from all 16 LN and three RCC patients were subjected to IMC spatial proteomic analysis using a panel of validated antibodies (**Table 3**).

**Table 1:** Demographics and clinical characteristics of a cohort of SLE patients.

Variable	SLE patients *n* = 16	Class IV LN *n* = 8	Class V LN *n* = 8	*p*-value^a^
Age (years)	48.0 (31.0–53.8)	37.0 (25.3–46.5)	53.0 (47.8–63.3)	0.027
Gender (female)	11 (68.8)	6 (75.0)	5 (62.5)	0.590
Ethnicity				0.522
Chinese	13 (81.3)	6 (75.0)	7 (87.5)	
Malay	3 (18.8)	2 (25.0)	1 (12.5)	
Disease duration (years)	0.0 (0.0–4.5)	0.0 (0.0–2.3)	0.5 (0.0–5.8)	0.452
Smoking status				0.584
Never-smoker	13 (81.3)	7 (87.5)	6 (75.0)	
Ex-smoker	2 (12.5)	1 (12.5)	1 (12.5)	
Current smoker	1 (6.3)		1 (12.5)	
Creatinine (µmol/L)	60.5 (50.5–90.3)	82.5 (61.8–102.3)	52.0 (46.3–60.8)	0.021
eGFR (mL/min)	100.0 (72.3–120.5)	90.0 (54.5–126.0)	110.0 (80.0–119.0)	0.917
UPCR (g/day)	2.2 (1.5–5.7)	2.7 (1.5–6.9)	2.2 (1.4–5.4)	0.916
First kidney biopsy	16 (100.0)	8 (100.0)	8 (100.0)	1.000
Indication for kidney biopsy				0.299
Initial diagnosis	13 (81.3)	7 (87.5)	6 (75.0)	
Kidney relapse	1 (6.3)		1 (12.5)	
Modified NIH activity index	4.5 (0.0–7.3)	7.0 (5.3–8.8)	0.0 (0.0–0.3)	<0.001
Endocapillary hypercellularity	1.0 (0.0–3.0)	3.0 (3.0–3.0)	0.0 (0.0–0.0)	<0.001
Neutrophil/karyorrhexis	0.0 (0.0–1.3)	1.0 (0.3–2.8)	0.0 (0.0–0.0)	0.010
Fibrinoid necrosis	0.0 (0.0–0.0)	0.0 (0.0–0.0)	0.0 (0.0–0.0)	0.386
Hyaline deposits	1.0 (0.0–2.3)	2.0 (1.0–3.0)	0.0 (0.0–0.0)	0.001
Cellular/fibrocellular crescents	0.0 (0.0–0.0)	0.0 (0.0–1.5)	0.0 (0.0–0.0)	0.170
Interstitial inflammation	0.0 (0.0–0.0)	0.0 (0.0–0.0)	0.0 (0.0–0.0)	0.285
Modified NIH chronicity index	3.0 (0.0–4.0)	1.0 (0.0–3.0)	4.0 (1.5–4.0)	0.127
Total glomerulosclerosis score	0.0 (0.0–1.0)	0.0 (0.0–1.0)	1.0 (0.0–1.5)	0.435
Fibrous crescents	0.0 (0.0–0.0)	0.0 (0.0–0.8)	0.0 (0.0–0.0)	0.779
Tubular atrophy	1.0 (0.0–1.0)	0.5 (0.0–1.0)	1.0 (0.0–1.0)	0.662
Interstitial fibrosis	0.0 (0.0–1.0)	0.0 (0.0–1.0)	1.0 (0.0–1.0)	0.463
Podocytopathy	2 (12.5)	0 (0.0)	2 (25.0)	0.131
Treatment				
Azathioprine	3 (18.8)	0 (75.0)	3 (37.5)	0.091
Cyclophosphamide	5 (31.3)	3 (37.5)	2 (25.0)	0.429
Cyclosporine	2 (12.5)	0 (75.0)	2 (25.0)	0.155
Mycophenolate mofetil/mycophenolate sodium	9 (56.3)	4 (50.0)	5 (62.5)	0.853
Rituximab	1 (6.3)	1 (12.5)	0 (87.5)	0.261
Tacrolimus	2 (12.5)	0 (75.0)	2 (25.0)	0.155

Data are frequency (%) or median (interquartile range).

^a^ Comparison between classes IV and V LN.

UPCR, urine protein creatinine ratio; NIH, National Institutes of Health.

**Table 2:** Demographics and clinical characteristics of SLE patients versus RCC controls.

Variable	RCC control *n* = 3	Class IV LN *n* = 8	Class V LN *n* = 8
Age (years)	49.0 (46.0–)	37.0 (25.3–46.5)	53.0 (47.8–63.3)
Gender (female)	3 (100.0)	6 (75.0)	5 (62.5)
Ethnicity			
Chinese	3 (100.0)	6 (75.0)	7 (87.5)
Malay		2 (25.0)	1 (12.5)
ROIs			
Total number of glomerular ROIs	6	26	21
Total number of tubulointerstitial ROIs	4	23	22
Response to therapy for LN			
CR		6 (75.0)	2 (25.0)
PR		1 (12.5)	3 (37.5)
NR		1 (12.5)	3 (37.5)

Data are frequency (%) or median (interquartile range).

ROIs, regions of interest; CR, complete response; PR, partial response; NR, no response.

**Table 3:** List of proteins interrogated by IMC.

Category	Markers
Resident renal cells	WT1, CD31, VCAM1, αSMA, aquaporin-1, NaKATPase, calbindin, PDGFRβ
Immune cells	CD3, CD4, CD8a, CD11c, CD45RO, CD20, CD163, CD68, CD14, CD15, CD16, CD56
Extracellular matrix proteins	Collagen I, collagen IV, fibronectin
Epithelial–mesenchymal plasticity regulators	S100A4, vimentin, β-catenin, integrin β1, integrin α2, E-cadherin
Functional status markers	HLA-DR, Ki-67, HIF1α, C5aR1, CXCR3, C3

The following targets could not be detected and were excluded from the analysis: CD3, CD8, CD11c, CD16, CD56, and PDGFRB.

### 3.2 Increased glomerular and tubulointerstitial immune infiltrates in LN kidneys compared to RCC controls

**Figure 1:**
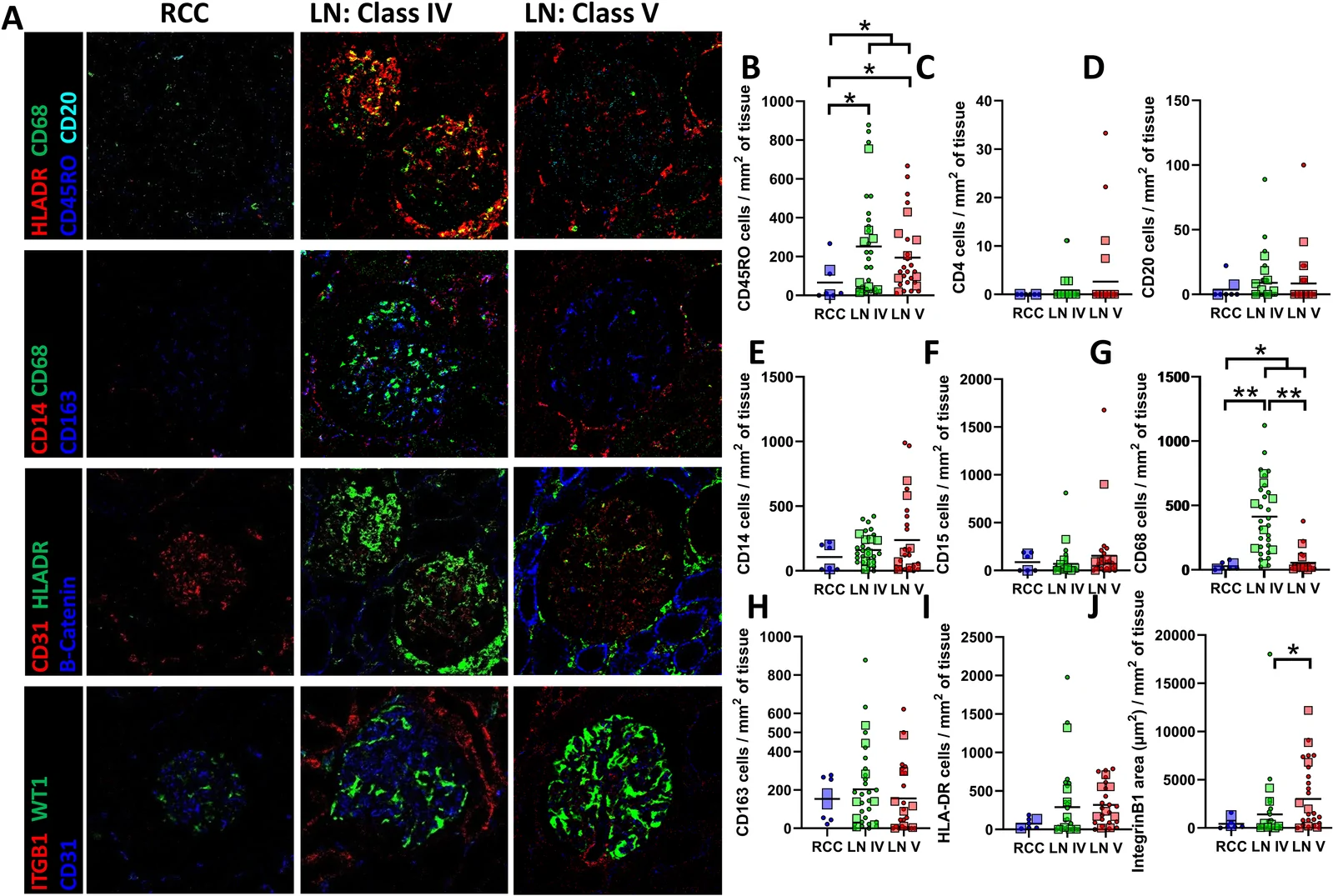
Increased glomerular immune cell infiltrates in LN kidneys compared to RCC controls. **(A)** In both class IV and class V LN biopsies, an increase in immune cell infiltrates inside the glomerulus and in the periglomerular regions was observed compared to the RCC controls. The IMC images shown are representative of 53 glomerular ROIs drawn from class IV LN patients, class V LN patients, and RCC controls. The targets displayed in this figure are shown pseudo-colored, as indicated. **(B–J)** Mann–Whitney *U*-tests were conducted to assess significant changes in glomerular infiltrating immune cell markers between class IV LN (green), class V LN (red), and disease controls (blue). Markers of immune cells, CD45RO (memory T cells), CD15, CD68, and CD163 (macrophages) and HLA-DR (antigen-presenting cells) were elevated in ROIs from LN biopsies compared to RCC controls. Whereas each circle represents a single interrogated ROI, the squares indicate the average at biopsy case level, and the line represents the mean in each group. *p < 0.05; **p < 0.01; ***p < 0.001.

**Figure 2:**
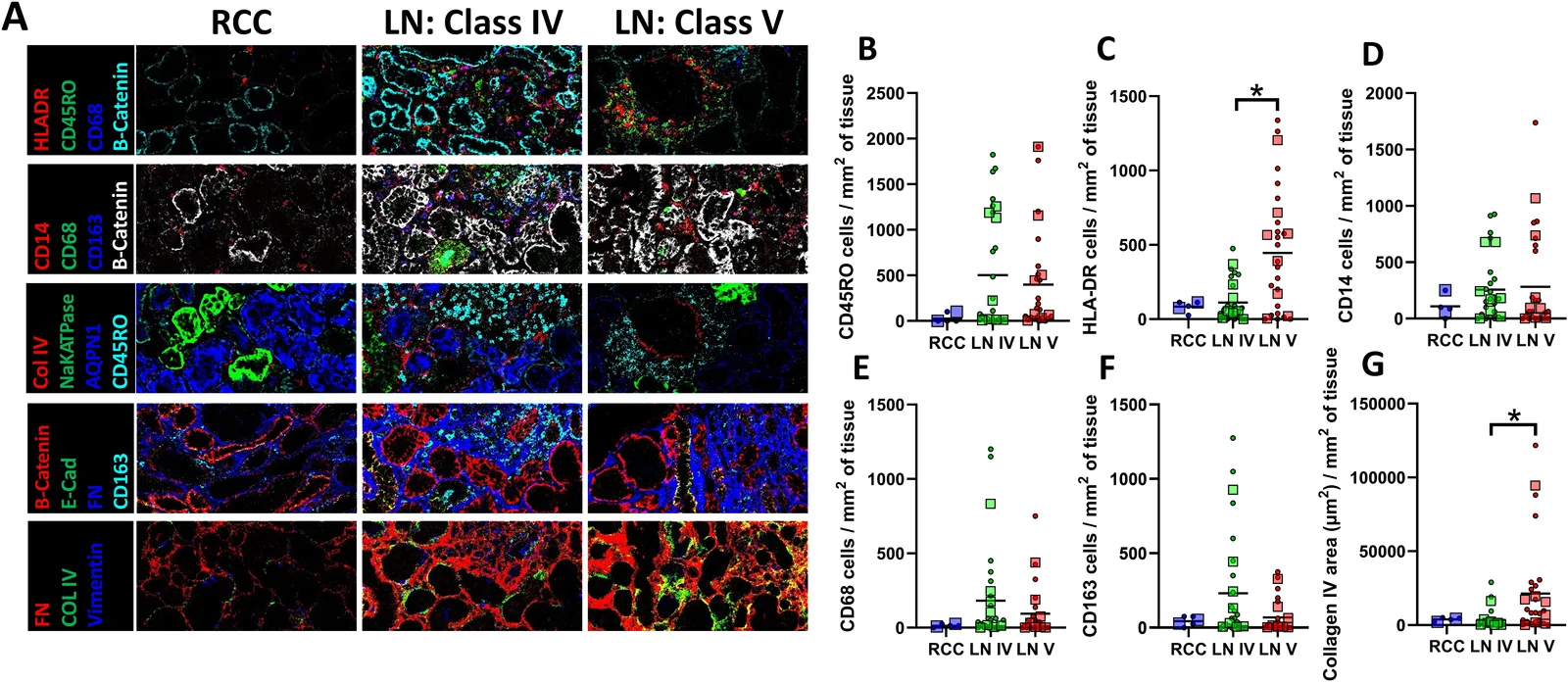
Increased tubulointerstitial immune cell infiltrates in LN compared to RCC controls. **(A)** The imaging mass cytometry images shown are representative of 49 tubulointerstitial regions of interest drawn from class IV LN patients, class V LN patients, and RCC controls. The 12 targets displayed in this figure are shown pseudo-colored, as indicated. **(B–G)** Mann–Whitney *U*-tests were conducted to assess significant changes in tubulointerstitial infiltrating immune cells between class IV LN (green), class V LN (red), and disease controls (blue). Whereas each circle represents a single interrogated ROI, the squares indicate the average at biopsy case level, and the line represents the mean in each group. In both class IV and class V LN biopsies, increased immune cell infiltrates in the tubulointerstitial regions were observed, including CD45RO (memory T cells), HLA-DR (antigen-presenting cells), and macrophages. *p < 0.05; **p < 0.01; ***p < 0.001.

**Figures 1** and **2** show the immune cell infiltration of the glomerular and tubulointerstitial ROIs from the LN and RCC kidneys. The markers used for IMC included antibodies to immune cells (CD45RO) such as B cells (CD20), T cells (CD3, CD4, CD8), macrophages (CD14, CD68, CD163), myeloid cells (CD11c, CD15, CD16), NK cells (CD56), and proliferating cells (Ki-67). Markers such as CD45RO (memory T cells), HLA-DR (antigen-presenting cells), and CD68 (macrophages) were increased in glomerular ROIs compared to RCC (**Figure 1**), while CD45RO and HLA-DR were increased in the tubulointerstitial ROIs compared to RCC (**Figure 2**). CD45RO expression was increased in class IV LN compared to class V LN in both glomerular and tubulointerstitial ROIs (**Figures 1B**, **2B**). Conversely, HLA-DR expression was increased in class V LN compared to class IV LN in both glomerular and tubulointerstitial ROIs (**Figures 1I**, **2C**). As CD68 is a pan-macrophage marker while CD45RO can also be expressed in activated/memory immune cells, co-staining analysis for CD45RO, CD68, and HLA-DR showed a strong expression of HLA-DR on the CD68^+^ macrophages, many of which were CD45RO^+^, particularly in the glomerular and periglomerular infiltrates of class V LN (**Figure 1A**). Taken together, this suggests that the glomerular and periglomerular macrophages of class V LN appeared to be activated (i.e., HLA-DR^+^ and CD45RO^+^). The images in **Figure 2** also underscore the significant tubular atrophy in LN (as marked by the smaller tubules separated by an expanded interstitium), with marked fibronectin deposition filling the interstitium. Interestingly, there was also significant interstitial collagen deposition in class V LN compared to class IV LN.

### 3.3 Increased glomerular/periglomerular collagen and fibronectin expression in class V compared to class IV LN

**Figure 3:**
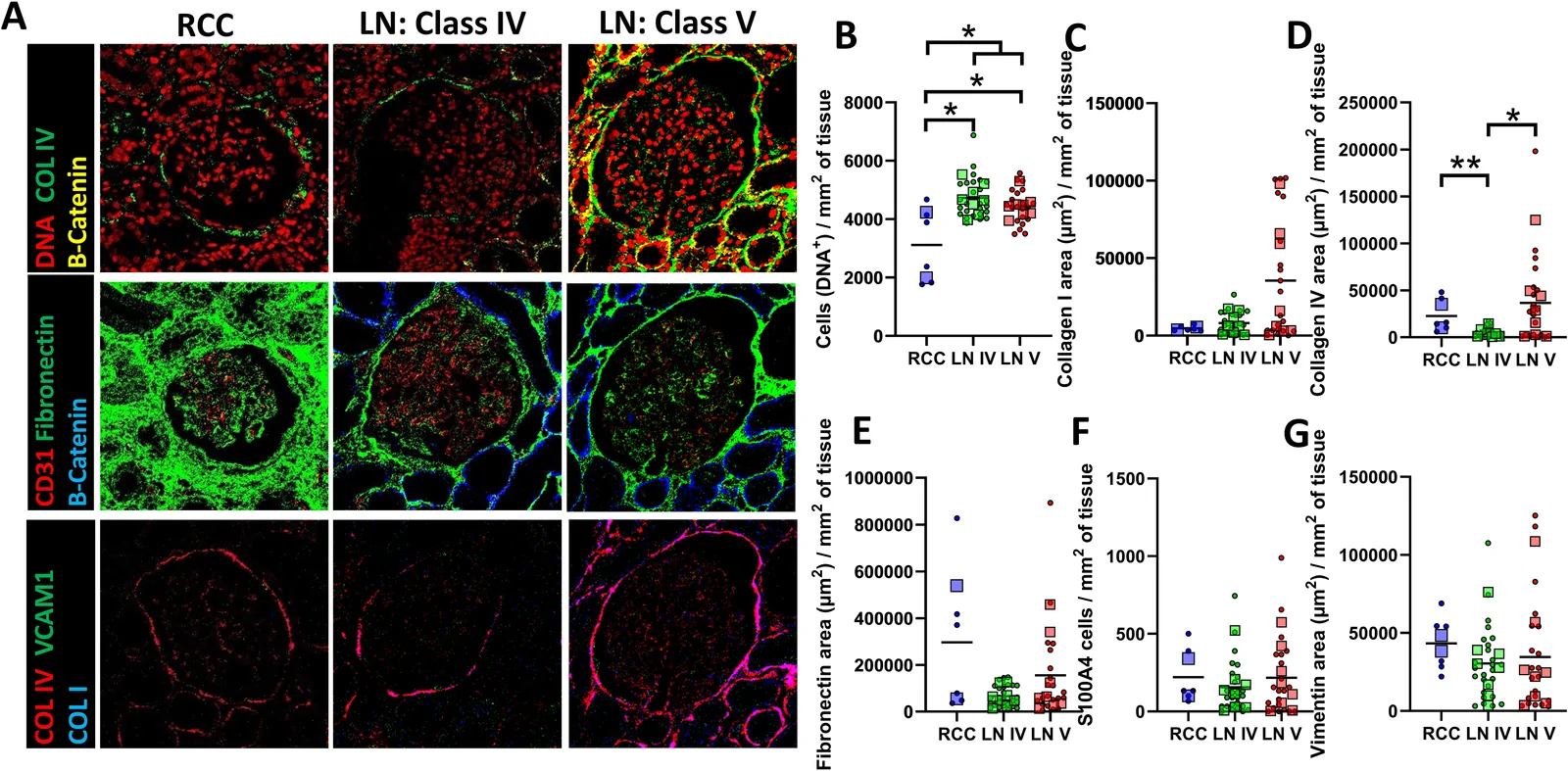
Increased glomerular/peri-glomerular collagen deposition and fibrosis are observed in class V LN compared to class IV LN. **(A)** The targets displayed in the imaging mass cytometry images are shown pseudo-colored, as indicated. The data shown are representative of 53 glomerular ROIs drawn from class IV LN patients, class V LN patients, and RCC controls. **(B–G)** Mann–Whitney *U*-tests were conducted to assess significant changes in glomerular fibrosis markers between class IV LN (green), class V LN (red), and disease controls (blue). Whereas each circle represents a single interrogated ROI, the squares indicate the average at biopsy case level, and the line represents the mean in each group. **(A, B)** Hypercellularity (DNA) in glomeruli was observed in class IV and class V LN biopsies compared to RCC controls. **(C–E)** In addition, kidneys with membranous LN (class V LN) showed increased periglomerular deposition of fibronectin, collagen I, and collagen IV. *p < 0.05; **p < 0.01; ***p < 0.001.

**Figure 4:**
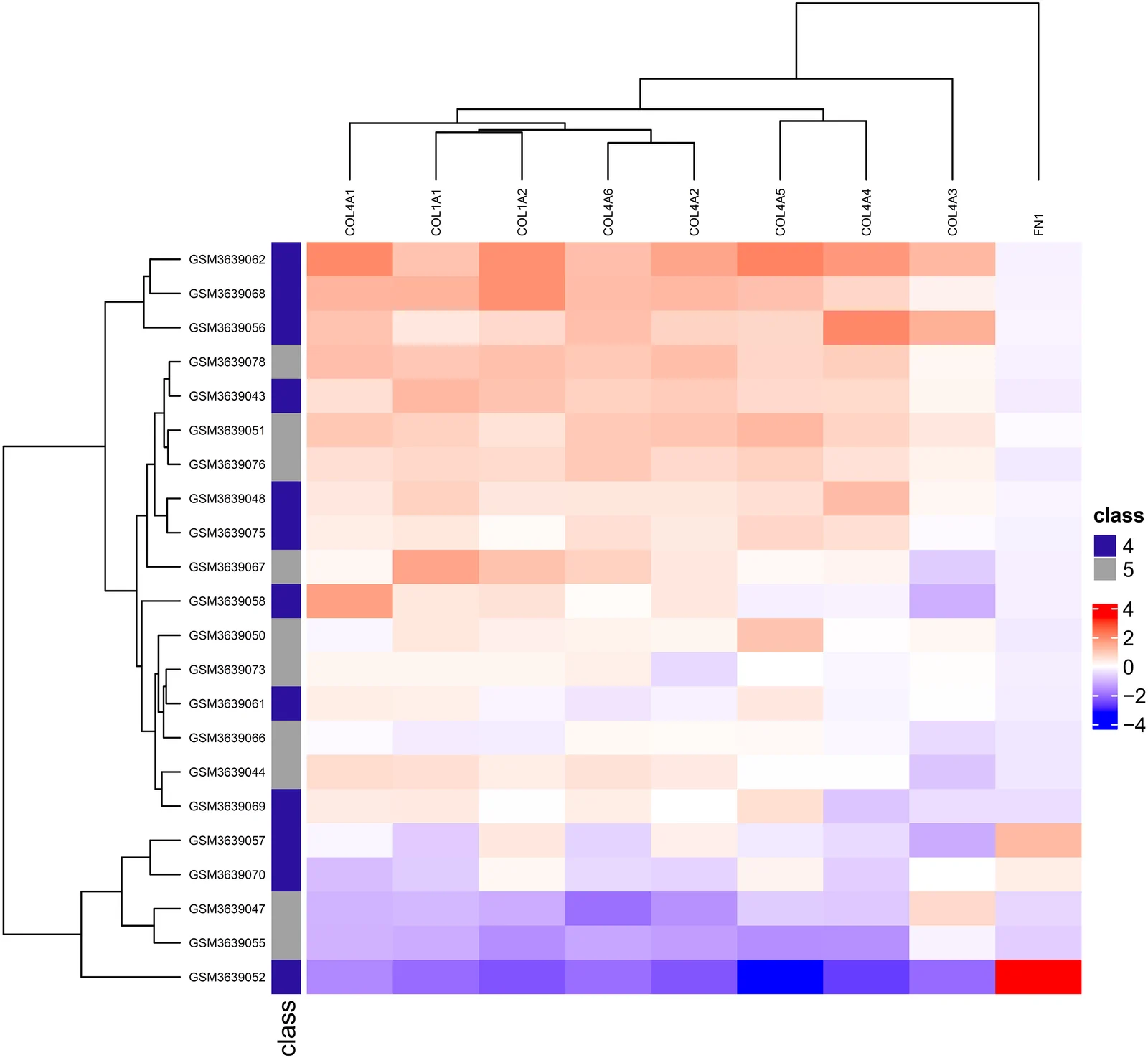
Fibrosis-related genes in micro-dissected glomeruli of patients with class IV and class V LN. Heatmap showing the hierarchical cluster analysis of fibrosis-related genes coding for collagen I, collagen IV, and fibronectin in the micro-dissected glomeruli of patients with class IV and class V LN. Color intensity scales from red (increased expression) to blue (decreased expression).

**Figure 3** shows glomerular/periglomerular collagen expression in class IV and class V LN. The images are pseudo-colored for collagen (collagen I and IV) and fibronectin. Class V LN exhibited significantly increased collagen I and IV staining compared to class IV LN (**Figures 3C, D**). Fibronectin staining was also increased in class V LN compared to class IV LN, along the periglomerular capsule, around intraglomerular vascular margins, and around the periglomerular tubules, although it did not reach statistical significance (**Figure 3E**). These three proteins (i.e., collagen I, collagen IV, and fibronectin) exhibited significant colocalization with each other (**Figure 3A**). In addition, increased fibronectin deposition was also noted in extracellular spaces outside the bounding membranes (**Figure 3A**). In contrast, in a publicly available mRNA expression dataset (GSE127797), none of the three fibrosis-related genes available in the microarray was differentially regulated in the micro-dissected glomeruli of patients belonging to class IV and class V LN (**Figure 4**) (13).

### 3.4 Identifying spatial proteomic predictors of poor response to immunosuppressive therapy in LN

**Figure 5:**
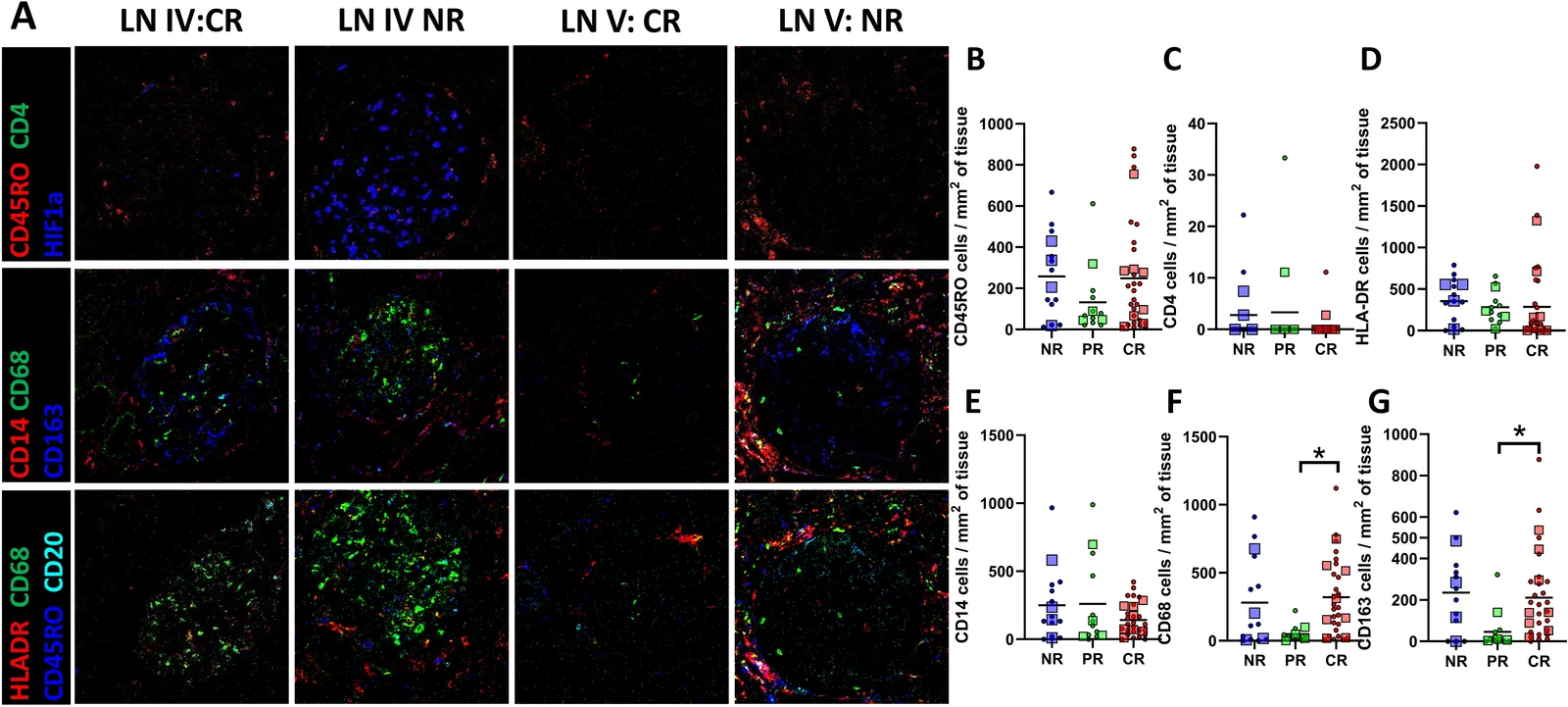
Glomerular immune cell infiltrates were increased in LN patients who did not respond to immunosuppressive therapy. **(A)** The imaging mass cytometry images shown are representative of 47 glomerular ROIs drawn from CRs, PRs, and NRs. The targets displayed in this figure are shown pseudo-colored, as indicated. **(B–G)** Mann–Whitney *U*-tests were conducted to assess significant changes in glomerular infiltrating immune cell markers between NRs (blue), PRs (green), and CRs (red). Whereas each circle represents a single interrogated ROI, the squares indicate the average at biopsy case level, and the line represents the mean in each group. Patients who did not respond to therapy (NRs) had significantly increased numbers of immune cell infiltrates into the glomeruli, including CD45RO^+^ memory T cells, CD4^+^ T cells, CD14^+^ macrophages, and CD163^+^ macrophages, compared to LN patients who responded to therapy (CRs and PRs). **(A–C, E, G)**. In patients who achieved CR, there was a significant reduction in the number of infiltrating HLA-DR^+^ antigen-presenting cells **(D)** compared to NRs and PRs. *p < 0.05; **p < 0.01; ***p < 0.001.

**Figure 6:**
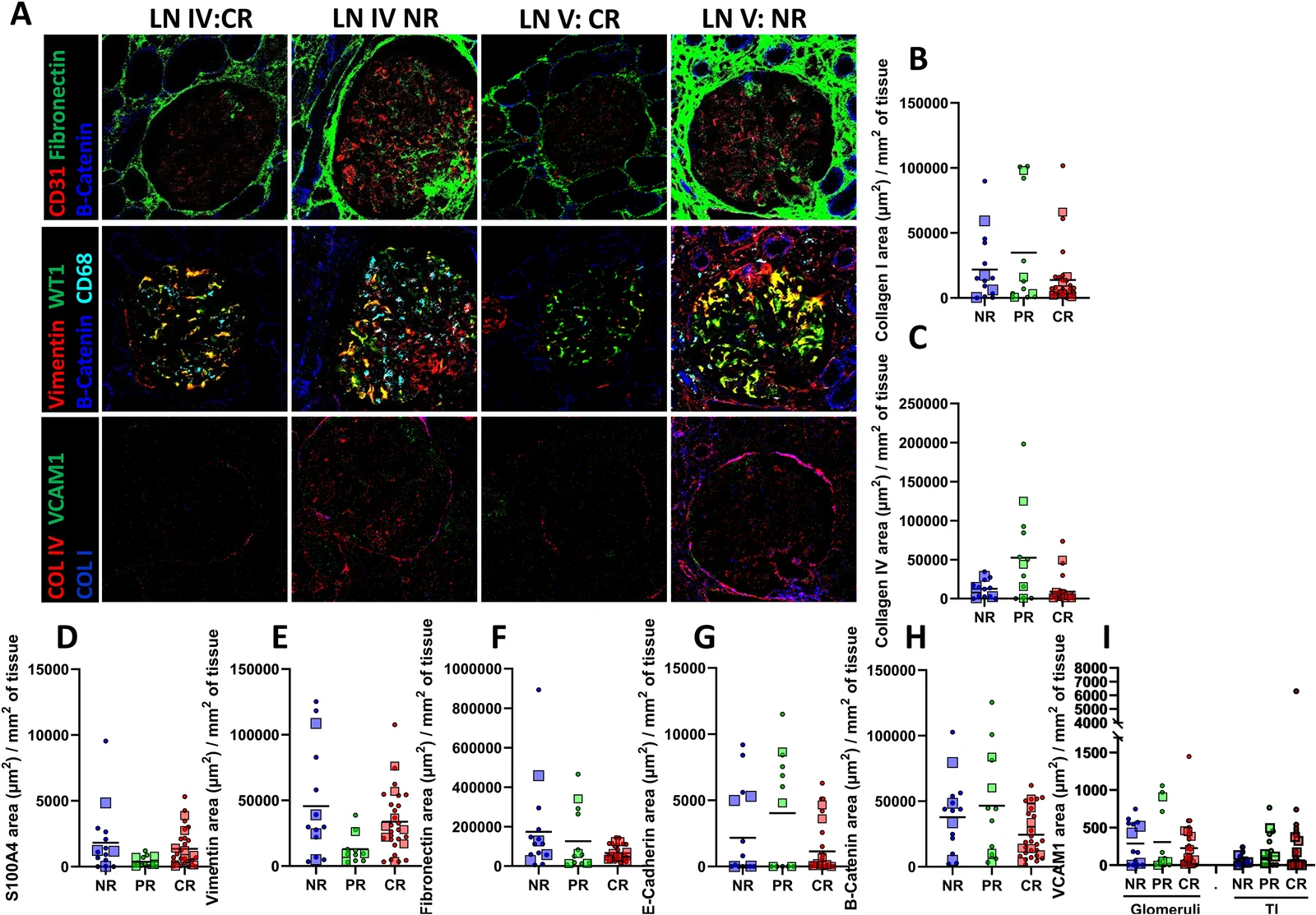
Pro-fibrotic markers were increased in the glomeruli of LN patients who did not respond to immunosuppressive therapy. **(A)** The imaging mass cytometry images shown are representative of 47 glomerular regions of interest drawn from CRs, PRs, and NRs. The targets displayed in this figure are shown pseudo-colored, as indicated. **(B–G)** Mann–Whitney *U*-tests were conducted to assess significant changes in glomerular pro-fibrotic markers between NRs (blue), PRs (green), and CRs (red). Whereas each circle represents a single interrogated ROI, the squares indicate the average at biopsy case level, and the line represents the mean in each group. **(A)** In LN patients who did not respond to immunosuppressive therapy, there was a significant increase in collagen I **(B)**, collagen IV **(C)**, S100A4 **(D)**, vimentin **(E)**, fibronectin **(F)**, E-cadherin **(G)**, B-catenin **(H)**, and VCAM-1 **(I)**. *p < 0.05; **p < 0.01; ***p < 0.001.

**Figure 7:**
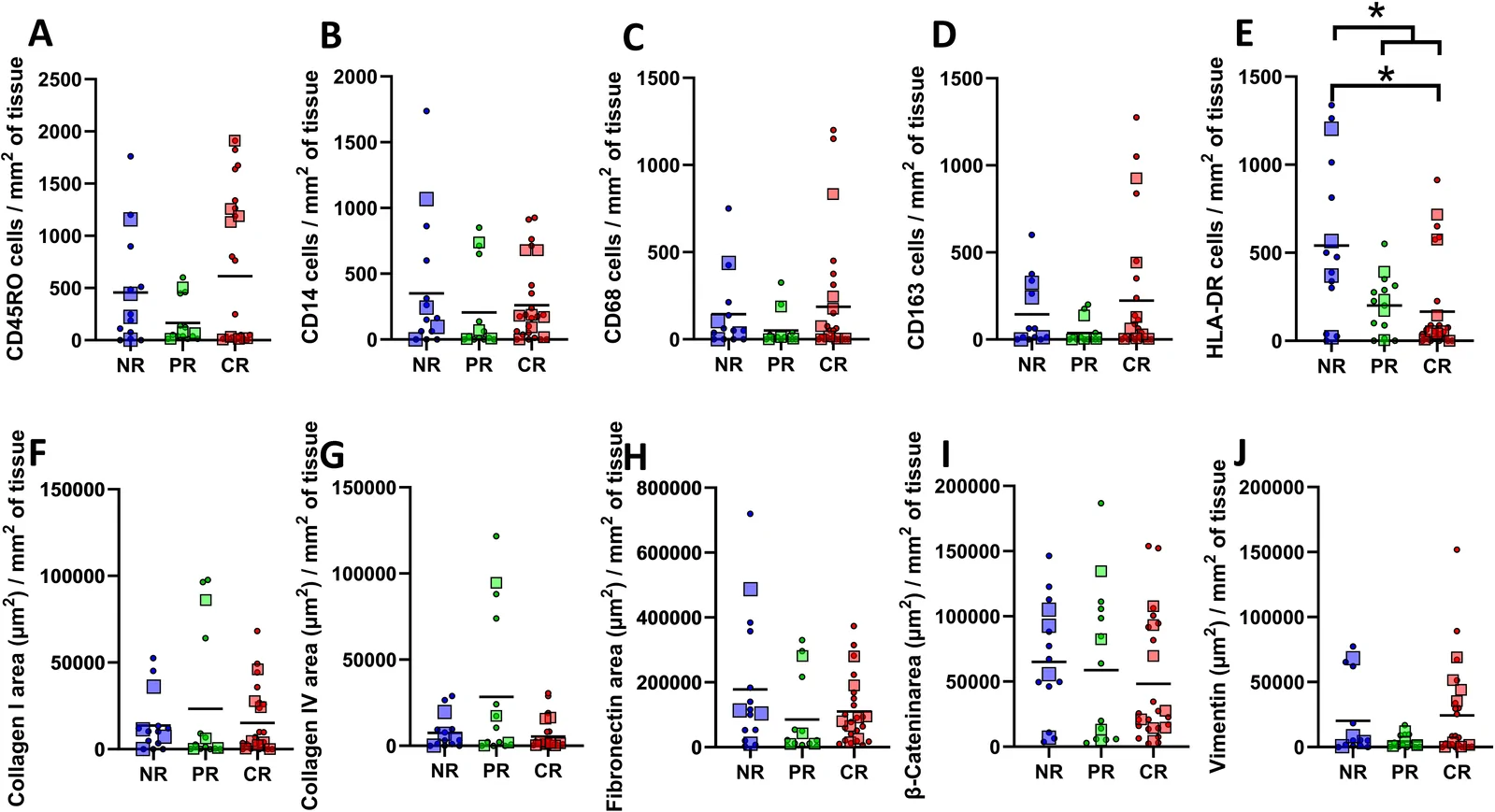
Tubulointerstitial inflammatory infiltrates, fibrosis, and EMP were increased in LN patients who did not respond to immunosuppressive therapy. Markers of antigen-presenting cells (HLA-DR), fibrosis (collagen I, collagen IV, and fibronectin), and EMP (B-catenin) in NRs compared to PRs and CRs. **(A–J)** Mann–Whitney *U*-tests were conducted to assess significant changes in tubulointerstitial infiltrating immune cell, pro-fibrotic, and EMP markers between NRs (blue), PRs (green), and CRs (red). Whereas each circle represents a single interrogated ROI, the squares indicate the average at biopsy case level, and the line represents the mean in each group. Patients who did not respond to therapy (NRs) had significantly increased HLA-DR and immune cells in the interstitium and peritubular margins and increased collagen I, collagen IV, and fibronectin expression, with prominent interstitial β-catenin deposition. *p < 0.05; **p < 0.01; ***p < 0.001.

**Figure 8:**
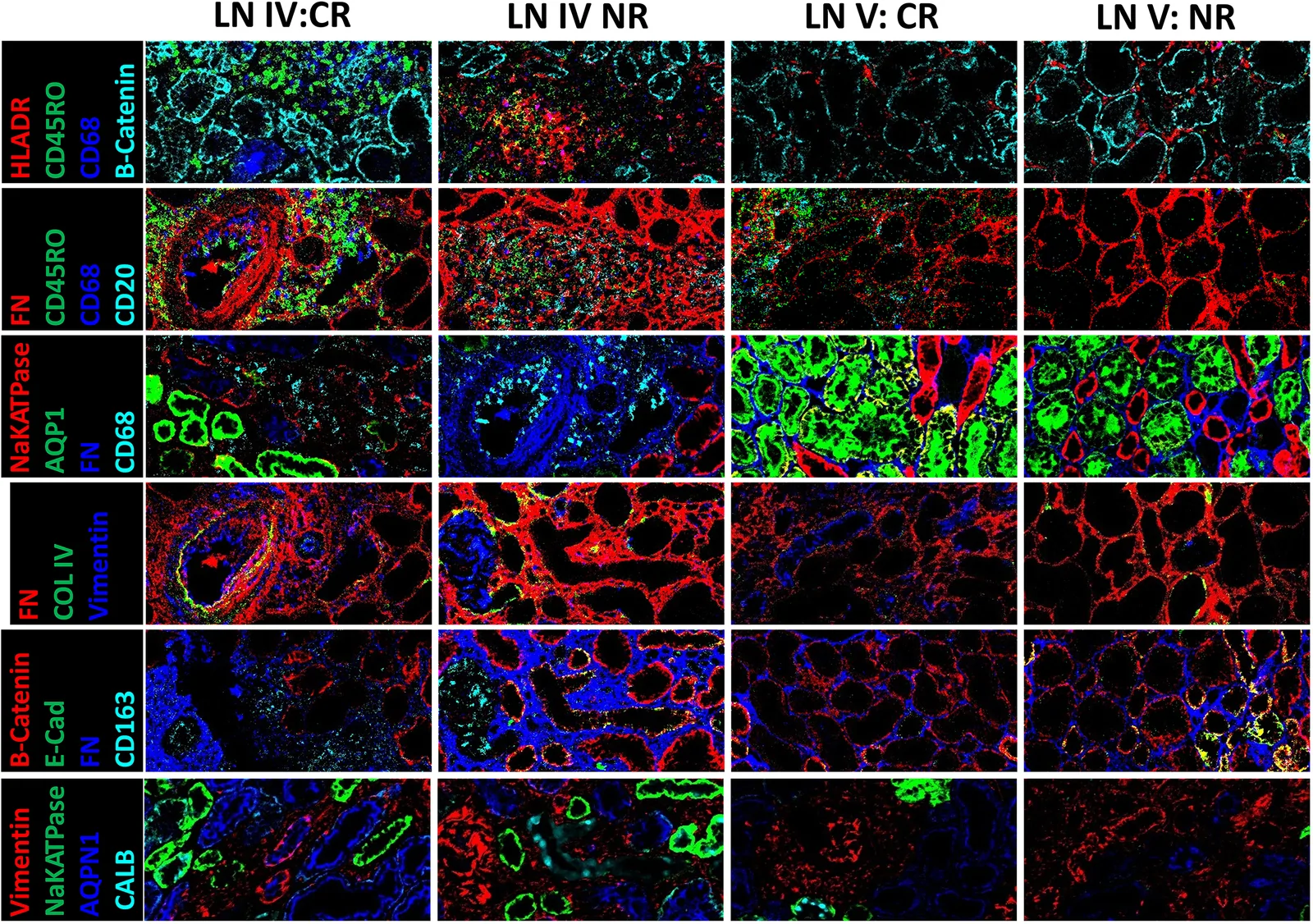
Imaging mass cytometry images of tubulointerstitial inflammatory infiltrates, fibrosis, and EMP in LN patients. Markers of antigen-presenting cells (HLA-DR), fibrosis (collagen IV), and EMP (B-catenin) in NRs compared to PRs and CRs. The imaging mass cytometry images shown are representative of 33 tubulointerstitial regions of interest drawn from CRs and NRs. The targets displayed in this figure are shown pseudo-colored, as indicated. Whereas each circle represents a single interrogated ROI, the squares indicate the average at biopsy case level, and the line represents the mean in each group.

At the time of kidney biopsy before treatment initiation, there were significantly increased markers of immune cell infiltrates and fibrosis in the glomeruli among LN patients found on follow-up to be non-responders (NRs) compared to complete responders (CRs) and/or partial responders (PRs). For this analysis, since the underlying LN class did not make a substantial impact on the outcome, both class IV and class V LN patients were pooled and then parsed by their treatment response status. The glomerular proteins that were increased among LN classes for NRs compared to CRs included CD45RO (memory T cells), CD4 (T cells), HLA-DR (antigen-presenting cells and memory T cells), CD14 (macrophages), CD163 (M2 macrophages), collagen I (fibrosis), collagen IV (fibrosis), and fibronectin (fibrosis) (**Figures 5**, **6**). Alongside these profibrotic changes, NRs also exhibited increased glomerular β-catenin, E-cadherin, and vimentin, markers of EMP (**Figure 6**). Parallel findings were documented in the tubulointerstitial regions, including increased HLA-DR and immune cells in the interstitium and peritubular margins and increased collagen I, collagen IV, and fibronectin expression, with prominent interstitial β-catenin deposition in NRs (**Figures 7**, **8**). In addition, there was a significant increase in tubular and parietal epithelial cell (PEC) expression (based on its unique spatial distribution) of the activation/injury marker VCAM-1 among NR (**Figures 6A, I**). The modified NIH chronicity index, but not modified activity index, was significantly higher in NRs and PRs compared to CRs (data not shown). We also observed highly significant positive correlations for the modified NIH activity index with hypoxia-inducible factor-1 alpha (*r* = 0.77, *p* < 0.001) and CD68 (*r* = 0.76, *p* < 0.001) (**Supplementary Table 1**).

### 3.5 Exploratory Bayesian network and ROI-level regression analyses

**Figure 9:**
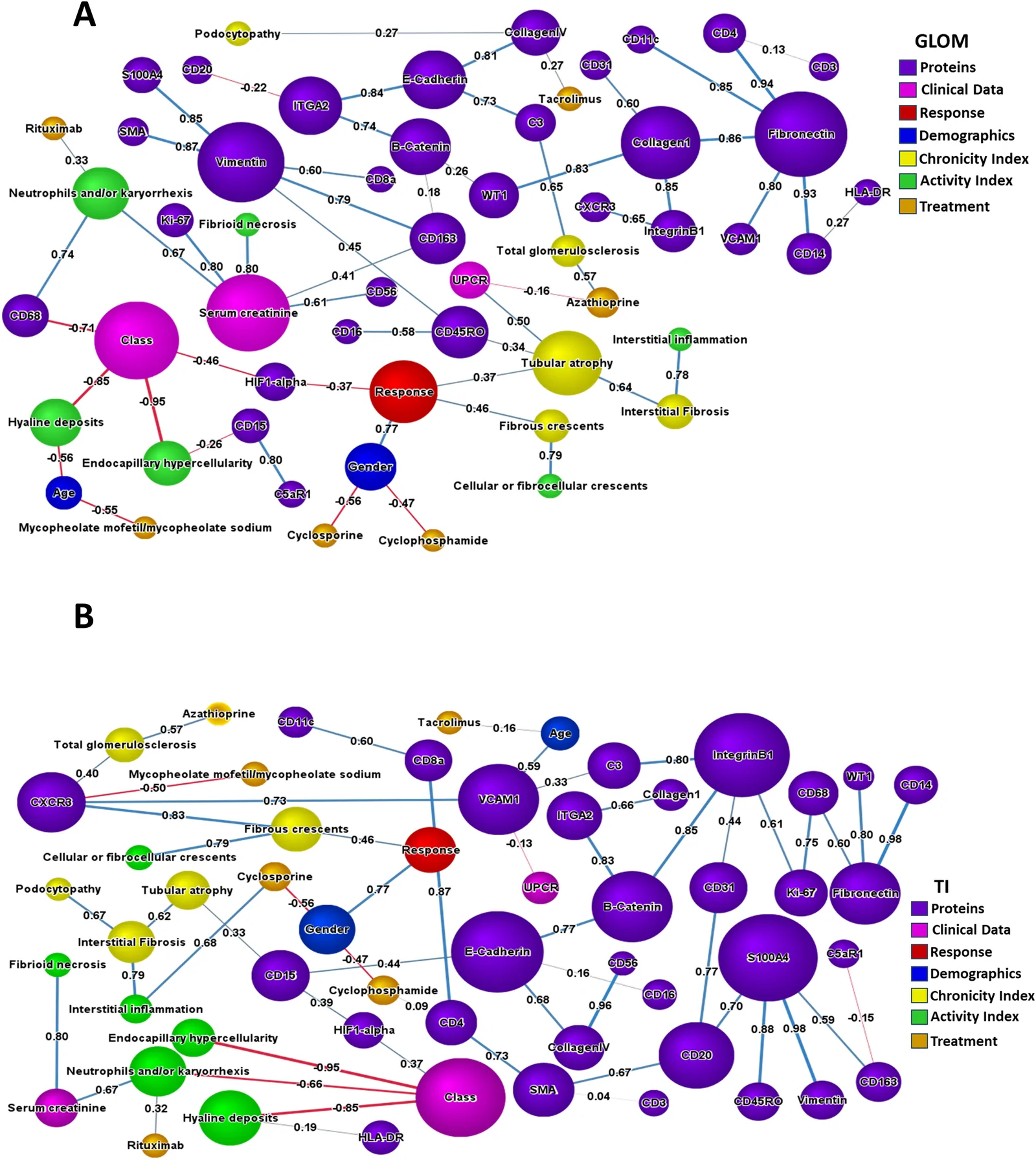
Exploratory Bayesian network analysis of spatial proteomic, clinical, pathological, and treatment-associated variables in lupus nephritis. Unsupervised Bayesian network analysis was performed using BayesiaLab to visualize probabilistic dependency networks associated with renal protein biomarkers expressed in the **(A)** glomerular and **(B)** tubulointerstitial compartments in lupus nephritis. The networks incorporated treatment outcomes, clinical variables, pathology indices, demographic features, treatment variables, and IMC-derived renal protein biomarkers from all subjects included in the study. Circular nodes represent proteins (purple), clinical features (red), demographic variables (blue), activity index variables (green), chronicity index variables (yellow), and treatment variables (orange). Node size was determined using “node force” and reflects the relative impact of each variable within the network based on conditional dependency relationships. Arcs between nodes represent probabilistic conditional dependencies identified by the Bayesian network algorithm. Arc thickness reflects the relative strength of the dependency, while arc color indicates the directionality of the Pearson correlation coefficient used for visualization purposes (blue = positive correlation; red = negative correlation).

Since IMC proteomics generated a high-dimensional dataset with a large number of spatial and clinical variables relative to the available cohort size, exploratory unsupervised Bayesian network machine learning analysis was performed to identify probabilistic dependency patterns among clinical, pathological, treatment, and IMC-derived variables rather than to develop a predictive model. As expected, renal pathology chronicity, glomerulosclerosis, fibrous crescents, and tubular atrophy exerted dominant effects on treatment response, while neutrophil karyorrhexis was most tightly associated with the myeloid cell marker CD68 (**Figure 9**), supporting the biological validity of this analytic approach. The treatment response node demonstrated associations with gender and fibrous crescents in both the glomerular and tubulointerstitial compartments, with non-response positively associated with male gender and increased fibrous crescents (**Figure 9**). In the glomerular network, fibronectin, collagen I, vimentin, and class of LN emerged as major network hubs demonstrating strong conditional dependencies with extracellular matrix remodeling, inflammatory, and injury-associated markers. An epithelial remodeling module consisting of E-cadherin, B-catenin, ITGA2, WT1, and collagen IV was also identified. Within the tubulointerstitial network, fibronectin, collagen I, VCAM1, IntegrinB1, and S100A4 formed prominent interconnected fibrosis-associated modules. Most interrogated inflammatory and fibrosis-associated protein markers, including CD45RO, CD163, fibronectin, and VCAM1, demonstrated strong interdependency patterns and collectively exerted major effects on tubular atrophy and interstitial fibrosis (**Figure 9**). To further evaluate ROI-level associations while accounting for pathology indices and treatment heterogeneity, multinomial regression analyses were performed separately for glomerular and tubulointerstitial ROIs. Modified NIH activity index, modified NIH chronicity index, and LN class were included as fixed adjustment variables in all models. Individual IMC biomarkers were evaluated in separate models together with one induction therapy variable at a time to minimize overfitting arising from the limited cohort size and high-dimensional proteomic dataset. Several nominally significant associations were identified (*p* < 0.05). In tubulointerstitial ROIs, increased S100A4, WT1, VCAM1, CXCR3, IntegrinB1, collagen IV, and SMA expression demonstrated positive associations with treatment response categories, whereas reduced ITGA2, B-catenin, and CD14 expression demonstrated associations with partial response groups. In glomerular ROIs, increased CXCR3, WT1, E-cadherin, IntegrinB1, and SMA expression also demonstrated significant associations with treatment response. Complete multinomial regression results are provided in **Supplementary Table 2**.

## 4 Discussion

We recruited a group of SLE patients for multiplexed spatial proteomics analysis to identify the baseline predictors of treatment response in class IV and class V LN. Several key findings were made in this study. First, there is increased glomerular and tubulointerstitial immune infiltrates in LN kidneys compared to RCC controls. Second, there was increased glomerular/periglomerular collagen deposition and fibrosis in class V compared to class IV LN despite similar chronicity indices. Third, glomerular, periglomerular, and tubulointerstitial immune infiltrates and fibrosis were increased in LN patients who did not respond to immunosuppressive therapy. The markers of nonresponse include CD45RO, HLA-DR, CD14 (macrophages), CD163 (M2 macrophages), collagen I, collagen IV, and fibronectin. Fourth, increased tubular and PEC expression of the activation/injury marker VCAM-1 also predicts treatment NR. Finally, an unsupervised Bayesian network analysis assessing all clinical and spatial proteomic variables revealed that gender, fibrous crescents, and chronicity index were the most central associations with treatment response in LN, while several of the immune and ECM markers associated with poor outcome were linked to tubular atrophy and interstitial fibrosis based on conditional probabilities.

Technologies like single-cell RNA sequencing (scRNA-seq) have delineated the cellular and molecular landscapes of LN by allowing for the quantification of gene expression at a single-cell level, enabling the identification of cell types and their associated gene expression profiles (14). To this end, two companion publications funded by the public–private Accelerating Medicines Partnership (AMP) program generated a single-cell atlas of human LN that described the cellular complexity of stromal and infiltrating leukocyte compartments (15, 16). The AMP SLE Network ran two parallel pipelines for the LN samples. One pipeline generated single-cell transcriptomes of total kidney cells, which was heavily represented by tubular cells (16). The separate pipeline specifically isolated CD45^+^ leukocytes by flow cytometric sorting, providing a high-resolution view of the infiltrating leukocytes in LN (15). A total of 21 immune cell subsets have been identified in LN kidneys through this approach, namely: four macrophage clusters, 10 clusters containing T cells or natural killer cells, two dendritic cell clusters, four B cell clusters, and one cluster of proliferating cells (15). The four clusters of macrophages include inflammatory CD16^+^ macrophages (CM0), phagocytic CD16^+^ macrophages (CM1), M2-like CD16^+^ macrophages (CM4), and tissue-resident macrophages (15). Trajectory analysis also identified a putative transition from inflammatory blood monocytes (CM0) to phagocytic macrophages (CM1) and then to alternatively activated macrophages (CM4) (15). The memory T cell populations include effector memory CD4^+^ T cells, central memory CD4^+^ T cells, and resident memory CD8^+^ T cells (15). In this study, we also reaffirm the prominence of memory T cells and macrophages in our IMC analysis with statistically significant increased markers of CD45RO, HLA-DR, and CD68 that were increased in ROIs of LN compared to RCC.

In the pipeline where scRNA-seq was performed for total kidney cells, genes encoding extracellular matrix (ECM) proteins and ECM-interacting proteins that are indicative of an active fibrotic pathway were enriched in the tubular cells of patients who did not respond to therapy (16). Four fibrotic genes—*COL1A1*, *COL14A1*, *COL1A2*, and *COL5A2*—predicted treatment response at 6 months after biopsy with an area under the curve of 0.96 (16). Using the tubular expression of fibrotic genes, a fibrosis score was created for each patient which was not different from classes IV and V LN (16). ECM proteins such as collagen I, collagen IV, and fibronectin were increased in non-responders for both glomerular and tubulointerstitial ROIs in our current study. Interestingly, we also observed increased glomerular/periglomerular fibrosis with increased collagen I, collagen IV, and fibronectin in class V LN compared to class IV LN. Tubular ECM protein expression has been linked to tubular EMP, a process whereby tubular cells differentiate into mesenchymal cells and begin secreting large amounts of ECM proteins (16). We also observed increased β-catenin in the tubulointerstitial space in this study. β-catenin is a well-established EMP-associated marker, and the Wnt/β-catenin signaling pathway is one of the main players in this process (17). We also identified increased CD163^+^ M2 macrophages in non-responders, and these pro-fibrotic macrophages have been associated with renal interstitial fibrosis and collagen deposition (17).

One of the important limitations of the aforementioned scRNA-seq data is that information on the anatomical location of the cells and their cellular interactions is lost when analyzing dissociated cells (14). Evaluation of glomerular and tubulointerstitial compartments separately provides spatial context for the transcriptomic findings (18). A recent study of LN utilizing a large gene expression panel to characterize the compartment-specific immune landscape via laser-capture microdissection showed that monocyte signatures arise mainly from the glomerular and periglomerular region, whereas T cell signatures are most abundant from the tubulointerstitial compartment (18). Per-protocol repeat kidney biopsies after completion of induction therapy showed that glomerular monocyte markers and tubulointerstitial T cell signaling differentiated non-responders from complete responders (18). Spatially resolved single-cell resolution spatial transcriptomics has recently been used to study childhood-onset LN, which represents a technical advance in the field (19). Our IMC data showed both increased CD45RO^+^ T cells and CD68^+^ macrophages in the glomerular region and predominant CD45RO^+^ T cells in the tubulointerstitial compartment. Although we did not have posttreatment per-protocol biopsies in this study, markers that reflect monocytes/macrophages and T cells such as CD14, CD68, CD163, and CD45RO were increased in non-responders in both glomerular and tubulointerstitial compartments in the diagnostic kidney biopsies performed during LN flare.

Major practice guidelines from the American College of Rheumatology, European League Against Rheumatism, European Renal Association–European Dialysis and Transplantation Association, and *Kidney Disease: Improving Global Outcomes* on the management of lupus nephritis have been published (3, 4, 20, 21). All of these major international guidelines advocated the use of the revised ISN/RPS classification to assess kidney biopsy in LN (3, 20, 22). The original NIH indices proposed by Austin et al. in 1983, comprising six activity scores and four chronicity scores, have a long development history (23). The revised ISN/RPS classification adopted the modified NIH indices to report overall activity and chronicity in a semiquantitative way (5). A future analysis to validate the modified NIH activity and chronicity indices using an evidence-based approach was proposed by ISN/RPS but has not been performed yet (5, 24). Initial validation by several groups have demonstrated that the modified chronicity index has a stronger correlation with kidney outcome (25–27)—for example, higher modified chronicity index, but not modified activity index, has been consistently identified as an independent predictor for poorer renal prognosis (25–27). The reason for this difference in utility might be due to the responsiveness of certain active lesions to aggressive immunosuppression (24). Our study demonstrated that both the modified chronicity index and fibrous crescent (a component of the modified chronicity index) were associated with treatment response, based on conditional probabilities, reaffirming the current view of the modified NIH indices. VCAM-1 is an adhesion molecule that plays an essential role in inflammation, being expressed in the inflamed vascular endothelium (28). In addition, non-vascular VCAM-1 expression in the kidney has also been recognized as an injury marker, both in renal tubules and in PECs (29). This is the first report revealing that the extent of tubular and PEC injury (as marked by VCAM-1 expression) is a predictor of non-responsiveness to immunosuppressive agents in LN. Older age at kidney biopsy has been reported to be correlated to the baseline chronicity index (30). This may explain the numerically higher modified NIH chronicity index observed in SLE patients with class V LN (**Table 1**). The observation that class V LN demonstrated increased collagen I, collagen IV, and fibronectin deposition despite comparable chronicity indices suggests that spatial proteomics may capture compartment-specific extracellular matrix deposition that is not fully reflected by conventional semiquantitative histologic scoring. The chronicity index summarizes established chronic lesions such as glomerulosclerosis, fibrous crescents, tubular atrophy, and interstitial fibrosis but may not fully capture the spatial distribution, intensity, or microanatomic localization of ECM protein accumulation. In our IMC analysis, the increased fibronectin and collagen signal was particularly evident in glomerular, periglomerular, vascular-margin, and peritubular regions, suggesting that membranous LN may be associated with localized matrix remodeling even when the overall chronicity score is not significantly different.

The limitations of this study include a relatively small sample size comprised of predominantly Chinese female LN patients from a single institution. Hence, the generalizability of our findings to other populations or healthcare settings is uncertain. The adjacent tissue in nephrectomy samples from RCC patients is not ideal as a control since peritumoral kidney often has molecular alterations with pathologic changes ranging from immune cell infiltrates (**Figure 1**), vascular sclerosis, interstitial fibrosis/tubular atrophy, and global glomerulosclerosis (31). A better control would be tissue from healthy persons obtained as part of living-donor kidney implantation biopsies (32). Induction therapy for LN was not standardized in our study and may introduce heterogeneity in the treatment responses. Due to the limited cohort size and high dimensionality of the spatial proteomic dataset, the regression analyses should be interpreted as exploratory and hypothesis-generating rather than definitive inferential models. In addition, due to the lack of long-term follow up data, we are unable to determine the longitudinal stability of the networks identified through machine learning. Upregulation of the fibrosis-related genes was not seen in the micro-dissected glomeruli of class V compared to class IV LN (**Figure 4**). This may be because the fibrosis changes were mainly in the periglomerular regions in our IMC data, while this publicly available microarray dataset was performed using micro-dissected glomeruli which might lose this spatial information. Moreover, ECM changes will be captured better by spatial proteomics if post-translational changes were at play. An additional technical limitation is that several IMC targets (CD3, CD8, CD11c, CD16, CD56, and PDGFRB) could not be reliably detected and were therefore excluded from downstream analysis, which limited our ability to fully resolve immune cell subsets. Although CD163 is commonly associated with alternatively activated or tissue-remodeling macrophages, CD163 expression alone is insufficient to define an M2 phenotype in human tissue. Future multiplexed studies with optimized antibody clones, incorporating complementary phenotypic and functional markers, additional controls, and complimentary spatial transcriptomic profiling will be needed to comprehensively define the cellular heterogeneity and spatial niches in LN. Lastly, low inter-pathologist concordance when determining classes and pathology indices may lead to the misclassification of LN and affect the current analysis. Nevertheless, this study also has its strength. This is the first study to utilize IMC proteomics and machine learning to dissect the heterogeneity of treatment response. Our study clearly illustrates the power of this approach to understand the immunopathology of treatment-resistant LN.

In summary, we have shown that through spatial proteomics that immune cell infiltrates, fibrosis, and VCAM-1 expression in kidney biopsies of class IV and class V LN differentiate NRs from CRs at time of flare. In particular, renal response is most closely related to the modified chronicity index which is, in turn, associated with activated M2 macrophages that induce fibrosis. We propose that such profibrotic macrophages have an important role in predicting renal prognosis since the current treatment armamentarium seems to be effective in the treatment of active lesions (17, 24). At present, no treatment can target macrophages, fibrosis, or tubular/PEC injury in LN. The improved understanding of macrophage interactions in LN may potentially contribute to developing new therapeutic pathways and strategies, which would be a major turning point in the treatment of SLE.

## Data Availability

The raw data supporting the conclusions of this article will be made available by the authors, without undue reservation.

## Supplementary material

Supplementary Table 1 Univariate correlation examining the association between modified NIH activity and chronicity indices with proteins interrogated by IMC.

Supplementary Table 2 ROI-level multinomial regression analysis of IMC biomarkers associated with treatment response after adjustment for modified NIH activity index, modified NIH chronicity index, LN class, and induction therapy.
