# Supplementary material for: T cell, M2 macrophage infiltration and collagen, fibronectin, VCAM-1 expression portend poor outcome in lupus nephritis
Source: Front Immunol. 2026 Aug 12;17:1802673. doi: 10.3389/fimmu.2026.1802673 (PMC13506827; doi:10.3389/fimmu.2026.1802673)
Supplement: Supplementary file 1 [file Table1.docx]

**Supplemental Table 1.** Univariate correlation examining the association between modified NIH activity and chronicity indices with proteins interrogated by IMC.
