# Supplementary material for: T cell, M2 macrophage infiltration and collagen, fibronectin, VCAM-1 expression portend poor outcome in lupus nephritis
Source: Front Immunol. 2026 Aug 12;17:1802673. doi: 10.3389/fimmu.2026.1802673 (PMC13506827; doi:10.3389/fimmu.2026.1802673)
Supplement: Supplementary file 2 [file Table2.docx]

**Supplemental Table 2.** ROI-level multinomial regression analysis of IMC biomarkers associated with treatment response after adjustment for modified NIH activity index, modified NIH chronicity index, LN class, and induction therapy.

| **Compartment** | **Biomarker** | **Treatment Adjusted** | **Response Comparison** | **Beta** | **OR** | **95% CI** | **p-value** |
| --- | --- | --- | --- | --- | --- | --- | --- |
| TI | S100A4 | Rituximab | CR vs NR | 3.22 | 25.06 | 2.22–283.10 | 0.009 |
| TI | S100A4 | Azathioprine | CR vs NR | 3.35 | 28.43 | 2.11–382.30 | 0.012 |
| TI | S100A4 | Tacrolimus | CR vs NR | 4.50 | 90.32 | 2.46–3318.94 | 0.014 |
| TI | CXCR3 | Azathioprine | CR vs NR | 5.77 | 320.91 | 3.14–32758.80 | 0.014 |
| TI | WT1 | Tacrolimus | CR vs NR | 7.36 | 1575.92 | 3.45–719990.70 | 0.018 |
| GLOM | CXCR3 | Rituximab | CR vs NR | 2.97 | 19.55 | 1.63–234.97 | 0.019 |
| GLOM | E-Cadherin | Azathioprine | CR vs NR | 2.14 | 8.51 | 1.42–51.16 | 0.019 |
| TI | VCAM1 | MMF | CR vs NR | 3.59 | 36.17 | 1.56–837.77 | 0.025 |
| GLOM | IntegrinB1 | Azathioprine | CR vs NR | 5.00 | 148.11 | 1.77–12416.19 | 0.027 |
| TI | CXCR3 | Tacrolimus | CR vs NR | 5.35 | 210.04 | 1.83–24155.28 | 0.027 |
| TI | CXCR3 | Rituximab | CR vs NR | 4.50 | 90.46 | 1.66–4935.40 | 0.027 |
| GLOM | CXCR3 | Cyclosporine | CR vs NR | 2.90 | 18.12 | 1.36–242.18 | 0.029 |
| GLOM | WT1 | Azathioprine | CR vs NR | 3.00 | 20.08 | 1.34–300.73 | 0.030 |
| TI | IntegrinB1 | Azathioprine | CR vs NR | 2.38 | 10.78 | 1.24–93.65 | 0.031 |
| TI | ITGA2 | Tacrolimus | PR vs NR | -5.59 | 0.004 | 0.00002–0.706 | 0.037 |
| TI | WT1 | Tacrolimus | PR vs NR | 6.27 | 529.18 | 1.39–201351.10 | 0.039 |
| TI | SMA | Cyclosporine | CR vs NR | 2.98 | 19.79 | 1.17–335.03 | 0.039 |
| GLOM | WT1 | Rituximab | CR vs NR | 2.51 | 12.30 | 1.13–133.79 | 0.039 |
| TI | CollagenIV | Azathioprine | CR vs NR | 2.79 | 16.25 | 1.06–248.06 | 0.045 |
| TI | B-Catenin | Rituximab | PR vs NR | -3.29 | 0.037 | 0.001–0.943 | 0.046 |
| GLOM | SMA | Rituximab | CR vs NR | 3.89 | 48.77 | 1.06–2236.14 | 0.046 |
| TI | SMA | Rituximab | CR vs NR | 3.02 | 20.40 | 1.05–397.73 | 0.047 |
| TI | CD14 | Tacrolimus | PR vs NR | -5.56 | 0.004 | 0.00002–0.982 | 0.049 |

OR, odd ratio
